# Draft Genome Sequence of *Ideonella* sp. Strain A 288, Isolated from an Iron-Precipitating Biofilm

**DOI:** 10.1128/genomeA.00803-17

**Published:** 2017-08-17

**Authors:** Burga Braun, Sven Künzel, Ulrich Szewzyk

**Affiliations:** a Technische Universität Berlin, Berlin, Germany; b Max Planck Institute for Evolutionary Biology, Plön, Germany

## Abstract

Here, we report the draft genome sequence of the betaproteobacterium *Ideonella* sp. strain A_228. This isolate, obtained from a bog iron ore-containing floodplain area in Germany, provides valuable information about the genetic diversity of neutrophilic iron-depositing bacteria. The Illumina NextSeq technique was used to sequence the draft genome sequence of the strain.

## GENOME ANNOUNCEMENT

A 16S rRNA gene sequence comparison of strain A 288 revealed a 98% similarity to *Ideonella dechloratans* (GenBank accession no. NR_026108) (1) by searching BLASTn (2) restricted to sequences from type material and 97% identity by EZBioCloud (3) searches. Phylogenetic analysis using the ARB software (4) by applying neighbor-joining analysis with the Jukes-Cantor distance model, including bootstrap resampling analysis for 1,000 replicates, revealed the undescribed *Comamonadaceae* bacterium MWH55 (accession no. AJ556799) as the closest neighbor. Determination of the most abundant taxon for the genome bin based on weighted scaffold length revealed 38.4% similarity to *Burkholderiales* bacterium JOSHI 001.

The genus *Ideonella* (1) belongs to the *Rubrivivax-Roseateles-Leptothrix-Azohydromonas-Aquincola-Ideonella* branch of the family *Comamonadaceae* (5). Currently, the genus *Ideonella* is composed of three valid published species, *I. dechloratans* (1), *I. azotifigens* (6), and *I. sakaiensis* (7), which were isolated from different habitats, such as activated sludge, a rhizosphere soil, and a wetland park. Only the genome of the poly(ethylene terephthalate) (PET)-degrading *Ideonella sakaiensis* strain 201-F6 (7) has been published. However, the whole-genome shotgun sequencing project of *Ideonella* sp. strain B508-1 has been deposited under accession number NZ_BADL00000000. Here, we present the third genome sequence of the *Ideonella* strain, isolated from novel habitat, i.e., a bog iron ore-containing floodplain area.

Strain A 288, which originated from an iron and manganese-depositing biofilm of the Lower Oder Valley National Park, was isolated and cultivated as described elsewhere (8). The iron-depositing ability of the strain, which formed dark, brown-colored colonies on iron- and manganese-containing media, was confirmed according to Schmidt et al. (8). Genomic DNA was extracted using the GeneMATRIX soil DNA purification kit (Roboklon, Berlin, Germany). The paired-end library was prepared according to the Illumina Nextera XT DNA library prep kit protocol. Genome sequencing was done on an Illumina NextSeq 500 sequencer using the NextSeq Mid-Output kit version 2 (300 cycles) by generating 21,664,180 raw reads. Demultiplexing was done with bcl2fastq version 2.18.0.12, and quality filtering of raw reads was performed using Trimmomatic version 0.36 (9). Reads were checked for ambiguous base calls and low complexity, employing the DUST algorithm (10), and filtered accordingly with an R script in Microsoft R Open version 3.3.2 (11), followed by preassembly with SPAdes version 3.10.0 (12) using default k-mer lengths up to 99 bp. Scaffolds of ≥500 bp of this preassembly were subject to extension and second-round scaffolding with SSPACE standard version 3.0 (13). Scaffolds of ≥2,500 bp were assigned to genome bins by MetaBAT version 0.32.4 (14), and functional annotation of draft genomes was performed with Prokka version 1.12b (15).

The draft genome included 164 contigs with an *N*_50_ assembly quality of 73,043 and *L*_50_ of 28. The shortest sequence was 2,506 bp, and the longest sequence was 327,546 bp. The total size of the draft genome was 6,980,783 bp, with a G+C content of 70%. Annotation resulted in 242 contigs, including 6,139 coding regions for 6,209 genes, 762 signal peptides, one clustered regularly interspaced palindromic repeat (CRISPR) unit, 2 rRNAs (16S and 23S), 56 tRNAs, 1 transfer-messenger RNA (tmRNA), 11 miscellaneous RNAs (miscRNAs), and 762 signal peptide-coding sequences.

### Accession number(s).

This whole-genome shotgun project has been deposited at DDBJ/ENA/GenBank under the accession number MWLO00000000. The version described in this paper is the first version, MWLO01000000.
